# Seismic behaviour analysis of a wind turbine tower affected by sea ice based on a simplified model

**DOI:** 10.1038/s41598-021-86142-0

**Published:** 2021-03-24

**Authors:** Shuai Huang, Qingjie Qi, Shufeng Zhai, Wengang Liu, Jianzhong Liu

**Affiliations:** 1 National Institute of Natural Hazards, Ministry of Emergency Management of China, Beijing, 100085 China; 2 China Coal Research Institute, Beijing, 100013 China; 3 Institute of Disaster Prevention, Sanhe, 065201 Hebei China

**Keywords:** Civil engineering, Energy infrastructure, Mechanical engineering

## Abstract

Ice-structure interaction threatens the safety of the offshore structure; however, dynamic seismic action even renders this process more sophisticated. This research constructed a simplified calculation model for the wind turbine tower, ice, and water under seismic loading, which could avoid solving the complex non-linear equations. Then, the seismic behaviour of the structure, *i.e.* wind turbine tower, in the presence and absence of influences of the sea ice was investigated, and we found the remarkable effect of sea ice upon the wind turbine tower when its mass is within a range; the wind turbine tower is found to have reduced capacity in energy dissipation, and thickness of tower walls or stiffening ribs is supposed to be enlarged for making the structure more ductile. Affected by the sea ice, the shear force and bending moment of the tower showed significant increases, and more attention needs to be paid to the tower bottom and action position of the sea ice. According to the dynamic similarity principle, finally paraffin was used to simulate sea ice, and shaking-table tests were performed for simulating dynamic ice-structure-water interactions. Results of shaking-table tests verified the rationality of our proposed simplified model.

## Introduction

North China belongs to the earthquake-prone area, and there is a four-month freeze every year in the sea. For example, the freezing range could reach thirty nautical miles, and its thickness could reach the 0.6 m. Sea ice affects dynamic responses of wind turbine towers to seismic actions; however, influences of earthquakes and sea ice upon the offshore structures are taken into account separately in most design specifications. For example, the codes including Canadian Standards Association code (CSA)^1^, Eurocode^2^, American Petroleum Institute code (API)^3^, et al. only point out that the sea ice load is an essential part to be considered in seismic design of structures, while none of them offers a calculation method to consider the dynamic interplays among ice, structure, and water under seismic actions. Ice load induced structural failure is one of the main disasters for the offshore structures; therefore, finding a reasonable computing method is urgent for seismic design of offshore structures.

Ice-structure dynamic interplay under seismic loading is not studied as extensively as the ice-structure interplay. For examples, Staroszczyk^4^, Yu et al.^5^, Sun et al.^6^, Paavilainen et al.^7^ and Brown et al.^8^ investigated the ice-structure interaction, and discussed on the structure-ice impact process; however, they did not mention ice-structure dynamic interplay under influences of seismic loading. It is considered that ice loading results from a complex process of the structure-ice interaction with lots of unknown factors^9^. Particularly, due to dynamic seismic actions, this process becomes more sophisticated. In addition, earthquakes can also induce hydrodynamic pressure. Jia and Han^10^ found that floating ice influences hydrodynamic pressure on structures. According to Kobayashi^11^, influences of sea ice are able to magnify hydrodynamic pressure, with the interaction effect non-ignorable. Yamauchi et al.^12^ investigated the effects of surrounding ice sheets on the design of foundations for gravity-based structures while an earthquake occurs, and found that the load combination of ice and earthquake depends on the indentation velocity and an interaction between structure and non-moving ice may mitigate the seismic load; Feng et al.^13^ found that the ice generated around the structure not only changes the constraints and boundary conditions of the structure, but also induces a greater force on the structure under seismic load; however, the effect of earthquake-induced hydrodynamic pressure is ignored in this study, and seismic dynamic behaviour of structures varied greatly if water-structure-ice dynamic interaction was considered simultaneously under earthquake action.

Although there are simplified approaches for calculating ice load available in design standards^14,15^, earthquake actions still bring difficulties when designing ice-structure dynamic interplays^16^. Kato et al.^17^ proposed a method using finite element modelling and the Monte Carlo simulation and offers a general procedure with which to evaluate the simultaneous ice loading in the design; Kouichi et al.^18^ simplified the structure and ice system to a three-particle system and investigated the effect of structural form on the internal force response of the interactions therein. Jia^19^ assessed the pier response to ice and earthquake actions through numerical analysis, and pointed out indicate that the analytical method proposed in this paper can provide references for the development of ice mechanics, and the method is feasible for the study of the response of deep-water piers. Using a new physical mechanism that links self-excited and forced vibrations, Xu et al.^20^ developed a model for dynamic ice-structure interplays of single degree-of-freedom. As mentioned above, plenty of works have been done for investigation of variations of methods for calculating the ice load. Among these researches, few studies could provide a simple calculation method for ice-structure-water dynamic interaction under seismic loading. In addition, few studies have been conducted into the seismic behaviour of wind turbines affected by sea ice; because such wind turbines are new structural forms, there is a lack of codified seismic design provision governing their analysis, especially for wind turbines located at sea.

In seismic design of offshore structures built in ice zones, it is necessary to deal with interplays of sea ice, water, and wind turbines. For such purpose, this paper explores an approach on simulating dynamic ice-structure-water interplays in response to seismic loading, and established a simplified model for a wind turbine tower subjected to ice load, hydrodynamic pressure, and seismic load; then we analysed the seismic behaviour of the structure in the presence and absence of influences from sea ice. Finally, shaking-table tests were conducted for verifying the rationality of the simplified model and the accuracy of the calculated results.

## Approach on simulating the ice-structure-water dynamic interaction

### Presentation of the approach

In our study, the effect of water upon the structure, that is, wind turbine tower, is mainly present as hydrodynamic pressure. Based on Morison’s hydrodynamic pressure theory^18^, water can be considered as added mass, while the hydrodynamic pressure shows equivalence to damping force and inertia force generated by relative motions of structures and added mass under earthquake actions. Dampers and springs with the same damping and inertia force are used to simulate damping and inertia force. Due to inspiration of the above ideas, the sea ice is also considered as the added mass, however, it is different from water. The sea ice is as an independent added mass, and the connection between the structure and the sea ice through the springs and dampers. The dynamic behaviour of the springs and dampers are simulated by the dynamic ice force model. The added mass used to simulate the sea ice is free at the direction for inputting seismic excitations while has constraints at other directions (Fig. S1). Based on the above idea, the calculation model which could consider the dynamic interaction of ice, structure, and water is built.

#### Calculation of hydrodynamic pressure

In accordance with the Morison’s theory^18^, Eq. (1) is used for calculating hydrodynamic pressure:

1 $$ f\left( t \right) = \left( {C_{M} - 1} \right)\rho \Delta V\ddot{x} + \frac{1}{2}C_{D} \rho A_{p} \dot{x}\left| {\dot{x}} \right| $$

where $C_{M}$, $C_{D}$, $\rho$, $A_{p}$ separately represent the inertia force coefficient; damping force coefficient; water density; area of the tower projected on the unit length; $\dot{x}$ and $\ddot{x}$ denote horizontal velocity and horizontal acceleration of the structure, respectively.

The term $\frac{1}{2}C_{D} \rho A_{p} \dot{x}\left| {\dot{x}} \right|$ is non-linear, thus the solution is difficult. We used a simplified method to solve it. First, it was supposed a constant relative velocity of two adjoining elements between the structure and water, with the simplified method shown in Eq. (2).

2 $$ \dot{x}\left| {\dot{x}} \right| = x_{rms} \sqrt {8/\pi } \dot{x} $$

where $x_{rms}$ represents the root-mean-square velocity.

Afterwards, we could calculate the equivalent additional mass of water by the Eq. (3).

3 $$ M_{ai} = \sum {\left( {C_{M} - 1} \right)\rho S_{ij} l_{ij} } $$

where $S_{ij}$ is the element area of the structure facing the water; $l_{ij}$ refers to one half of effective length for element *ij*.

Finally, earthquake-induced hydrodynamic pressure could be calculated by substituting Eqs. (2) and (3) into Eq. (1).

#### Determining ice load

**Figure 1:**
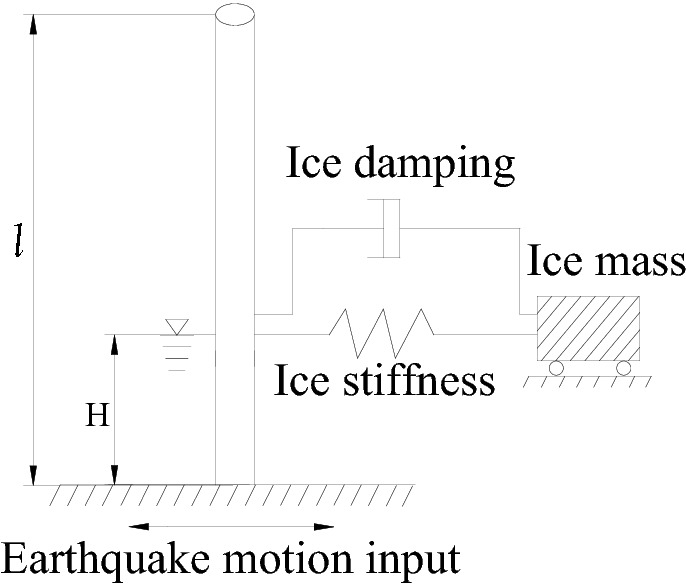
Calculation method of dynamic interplays between ice and structure.

Based on the above ideas, mass points and springs are used to take effects of free sea ice upon the structure, as shown in Fig. 1.

The research considers sea ice load on the tower by using added mass and springs, with stiffness of springs following the ice force model suggested by Croteau (Fig. S2), in which Korzhavin’s method is used for computing the highest ice force^19^. Persistent ice-breaking force $F_{{{\text{con}}}}^{c}$ is one third of the maximum ice force $F_{\max }^{c}$. The relative displacement $u_{2}$ = 2 $u_{1}$, and the relative displacement in the model are critical influencing factors in the model; whereas, no theoretical or empirical formulas are available to analyse or solve the ice force model. Therefore, parameters for relative displacement in the model, together with persistent ice-breaking force, are attained through ice-sheet penetrating experiments applying parameters the same as those used in numerical simulations.

#### Determining wind load

Wind load acts on particles through concentrated force, and its standard value is computed following the current code^20^, see Eq. (4).

4 $$ \omega_{ki} = \beta_{zi} \mu_{s} \mu_{zBi} \omega_{0} $$

where ${\omega }_{ki}$ and ${\omega }_{0}$ are the standard value of wind load on unit projected area at height *z* on the tower and the basic wind pressure, which is 0.7 kN/m^2^ for the 50-year return period following the code^21^; $\mu_{zBi}$ denotes the height-varying factor of wind load at *z* on the tower; $\mu_{s}$ and $\beta_{zi}$ separately represent the shape coefficient of wind load and the wind fluttering factor at *z* on the structure.

### Dynamic equilibrium in the simplified calculation model

The acceleration input model can be used for time-history analysis for the simplified model, which considers hydrodynamic pressure and sea ice as added mass. Equation (5) can be used to calculate the wind turbine tower of a single pile under seismic actions.

5 $$ [M]\{ \ddot{x}\} + [C]\{ \dot{x}\} + [K]\{ x\} = - [M]\{ \ddot{y}\} - [K_{Ds} ]\{ \dot{x}\left| {\dot{x}} \right|\} - W $$

where $$M$$,$$ C$$,$$ K$$, $$x$$, and $$\ddot{y}$$ represent the mass matrix, damping matrix, stiffness matrix, relative displacement of structural responses, and seismic acceleration, respectively; $$K_{Ds}$$ denotes the drag coefficient, $$K_{Ds} = \frac{1}{2}C_{D} \rho A_{p}$$; $$W $$ is the wind load (taken to be constant).

$M_{jk}$ is the position where sea ice acts on the structure, and Eq. (6) can be used to calculate the dynamic equilibrium for *M*_*jk*_:

6 $$ \begin{aligned} & \left[ {\begin{array}{*{20}c} {M_{i} + M_{ai} } & 0 & 0 & 0 & 0 \\ {} & {M_{jm} + M_{ajm} } & 0 & 0 & 0 \\ {} & {} & {M_{jk} + M_{ajk} } & 0 & 0 \\ {} & {} & {} & {M_{jn} + M_{ajn} } & 0 \\ {sym} & {} & {} & {} & {M_{u} } \\ \end{array} } \right]\left\{ {\begin{array}{*{20}c} {\ddot{x}_{i} } \\ {\ddot{x}_{j1} } \\ {\ddot{x}_{jk} } \\ {\ddot{x}_{jn} } \\ {\ddot{x}_{u} } \\ \end{array} } \right\}{ + }\left[ {\begin{array}{*{20}c} {C_{i} } & { - C_{i} } & 0 & 0 & 0 \\ {} & {C_{jm} + C_{jm} } & { - C_{jk} } & 0 & 0 \\ {} & {} & {C_{i} + C_{jk} + C_{k + 1} } & { - C_{k + 1} } & 0 \\ {} & {} & {} & {C_{jn} + C_{u} } & { - C_{u} } \\ {sym} & {} & {} & {} & {C_{u} } \\ \end{array} } \right]\left\{ {\begin{array}{*{20}c} {\dot{x}_{i} } \\ {\dot{x}_{j1} } \\ {\dot{x}_{jk} } \\ {\dot{x}_{jn} } \\ {\dot{x}_{u} } \\ \end{array} } \right\} \\ & \quad + \left[ {\begin{array}{*{20}c} {K_{i} } & { - K_{i} } & 0 & 0 & 0 \\ {} & {K_{jm} + K_{jm} } & { - K_{jk} } & 0 & 0 \\ {} & {} & {K_{i} + K_{jk} + K_{k + 1} } & { - K_{k + 1} } & 0 \\ {} & {} & {} & {K_{jn} + K_{u} } & { - K_{u} } \\ {sym} & {} & {} & {} & {K_{u} } \\ \end{array} } \right]\left\{ {\begin{array}{*{20}c} {x_{i} } \\ {x_{jm} } \\ {x_{jk} } \\ {x_{jn} } \\ {x_{u} } \\ \end{array} } \right\} = - \left[ {\begin{array}{*{20}c} {M_{i} + M_{ai} } & 0 & 0 & 0 & 0 \\ {} & {M_{jm} + M_{ajm} } & 0 & 0 & 0 \\ {} & {} & {M_{jk} + M_{ajk} } & 0 & 0 \\ {} & {} & {} & {M_{jn} + M_{ajn} } & 0 \\ {sym} & {} & {} & {} & {M_{u} } \\ \end{array} } \right]\left\{ {\begin{array}{*{20}c} 1 \\ 1 \\ 1 \\ 1 \\ 1 \\ \end{array} } \right\}\ddot{y} \\ & \quad - \left[ {\begin{array}{*{20}c} {K_{Di} } & 0 & 0 & 0 & 0 \\ {} & {K_{Dsm} } & 0 & 0 & 0 \\ {} & {} & {K_{Dsk} } & 0 & 0 \\ {} & {} & {} & {K_{Dsn} } & 0 \\ {sym} & {} & {} & {} & 0 \\ \end{array} } \right]\left\{ {\begin{array}{*{20}c} {\dot{x}_{i} \left| {\dot{x}_{i} } \right|} \\ {\dot{x}_{jm} \left| {\dot{x}_{jm} } \right|} \\ {\dot{x}_{jk} \left| {\dot{x}_{jk} } \right|} \\ {\dot{x}_{jn} \left| {\dot{x}_{jn} } \right|} \\ {\dot{x}_{u} \left| {\dot{x}_{u} } \right|} \\ \end{array} } \right\} - \left\{ {\begin{array}{*{20}c} 0 \\ {W_{jm} } \\ {W_{jk} } \\ 0 \\ {W_{u} } \\ \end{array} } \right\} \\ \end{aligned} $$

where subscript *i* represents a term for sea ice; subscripts *j*, *u*, and *a* are terms for the wind turbine tower, superstructure concentration particles, and added mass; subscripts *m* and *n* represent terms before and after the action point *k* of ice and structure; $x_{i}$, $x_{j}$, and $x_{u}$ denote relative displacements of sea ice, mass points of the structure, and superstructure; $W_{u}$, $W_{jm}$, and $W_{jk}$ are equivalent static wind load on the top, middle, and bottom of the structure above the action position of sea ice, respectively.

Because water and ice are simplified to concentrated mass in our proposed simplified calculation model, the combination of ice, structure, and water is regarded as a multi-mass elastic system, and there will be no need to establish a 3D model for the structure. Also, the model does not involve the element grid, and lots of nonlinear nonconvergence problems are avoided. There are lots of numerical approaches for solving the dynamic equilibrium equation for this simplified calculation model, and Newmark-*β* method is widely used at present. This method is easy to converge and it has many advantages^22^. Therefore, the Newmark-*β* method is applied for solving the dynamic equilibrium equation for this simplified calculation model in our study.

## Numerical example

### Calculation model

**Figure 2:**
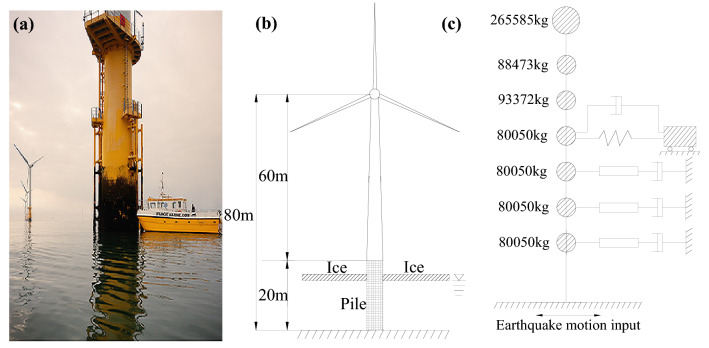
Diagram for a monopole wind turbine tower: (**a**) actual model; (**b**) geometric model; (**c**) simplified model.

A monopole wind turbine in the Yellow Sea was used in this study, where it is close to the coast of Jiangsu province in China and at 8-degree seismic fortification intensity according to the new seismic ground motion parameters zonation map of China^21^. This is a seismically active area (Class-II) where water is 15 m deep. The tower is 60 m high, and length of the pile is 60 m as well, with a 20-m-high above-ground part and a 40-m-high part underground. The wind turbine tower has a cylindrical structure with varying cross section and varying wall thickness; the tower and above-ground pile are manufactured with Q345C steel. The structure has an outer diameter ranging from 3.1 to 4.5 m and wall thickness from 18 to 50 mm. Poisson ratio, yield strength, and elasticity modulus of the material are *γ* = 0.3*σ*_*y*_ = 345 MPa, and *E* = 2.06 × 10^11^ Pa, respectively. To adopt the proposed simplified calculation model, the mass of the structure was ascertained in Fig. 2.

The bilinear model that is recommended by the Japanese *Standard Specification for Highway Bridges* is used as the stress–strain relationship of steel^23^, simplifying the plasticity stage and enhancing the material stage into a diagonal line (Fig. S3). Correspondingly, the elastic modulus is *E*/100. Table 1 lists parameters for bending capacity at bottom section of the structure.

**Table 1:** Parameters for bending capacity.

Physical quantity	Yielding moment $$M_{y}$$$$({\text{MN}}\;\;{\text{m}})$$	Yield curvature $$\phi_{y}$$$$\left( {{\text{m}}^{ - 1} } \right)$$	Limit bending moment $$M_{u}$$$$({\text{MN}}\;\;{\text{m}})$$	Ultimate curvature $$\phi_{u}$$ $$\left( {{\text{m}}^{ - 1} } \right)$$	Curvature ductility factor $$\phi_{u} /\phi_{y}$$
Values	30.6	0.0007	40.56	0.0146	15.87

### Selection of seismic waves

**Figure 3:**
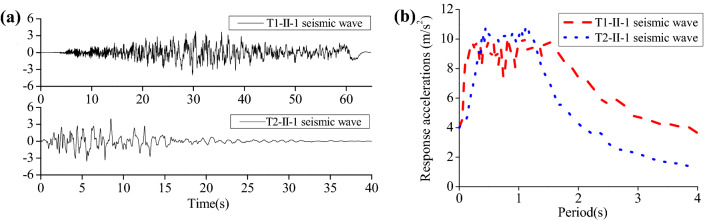
Seismic waves and its response spectrum: (**a**) acceleration time histories; (**b**) response spectrum.

There's mainly Subei coastal fault in this region and a total of 57 destructive earthquakes were recorded, and the maximum magnitude of earthquake ever recorded was 8 degrees. According to the Chinese code^24^, in seismic design, crucial structures are supposed to be increased by one degree. Therefore, the current research designed the seismic fortification intensity as 9, in which horizontal peak acceleration was 4 m/s^2^. According to the Japanese code^23^, far-field and near-field seismic waves (Site Type-II) were employed, separately termed as T1 and T2. The seismic waves and response spectrum with a damping coefficient of 0.05 were calculated (Fig. 3).

As seen from Fig. 3, the far-field seismic wave T1-II-1 exhibits the largest excellent platform, which is followed by near-field seismic wave T2-II-1. Responses of the structure with a natural vibration period between 0.25 and 1.6 s are remarkably influenced by the far-field seismic wave T1-II-1, with slowly decreasing acceleration responses if the structure has a natural vibration period exceeding 1.6 s. Responses of the structure with a natural vibration period from 0.4 to 1.2 s are greatly affected by the near-field seismic wave T2-II-1, with faster decline of acceleration responses than the far-field one T1-II-1.

### Seismic behaviours of the wind turbine tower

**Figure 4:**
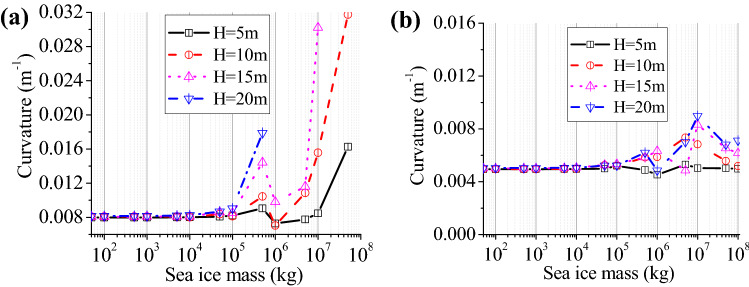
Highest curvature of the structural bottom at various water depths: (**a**) T1-II-1; (**b**) T2-II-1.

Different water depths will directly lead to the different action point of the sea ice upon the wind turbine tower; therefore, we investigated effects of ice thickness and water depth upon non-linear seismic behaviours of the structure. In aseismic design, the structural curvature was conveniently used in analysis of nonlinear behaviours, so we applied the highest curvature at bottom of the structure for investigating non-linear behaviours of the structure. The average thickness of the ice is 0.6 m, while the water is set to have depths of 5, 10, 15, and 20 m, respectively. For the proposed simplified model, sea ice was reduced to lumped mass, and the change laws of the maximum curvature with sea ice mass increasing are shown in Fig. 4.

As shown in Fig. 4, the highest curvature in response to T1-II-1 has a larger value than that to T2-II-1. With mass within a range, sea ice greatly influences the wind turbine tower; when the ice mass is varied from 0 kg to 1 × 10^8^ kg, the maximum curvature will exceed the ultimate curvature of the structure under T2-II-1, indicative of damage of the structure. The mass of sea ice corresponding to the structure damage decreases with the increasing water depth. For example, the curvature reaches a maximum of the mass of sea ice is 5 × 10^7^ kg in water depths 5 m, and the curvature reaches the maximum if sea ice has the mass of 5 × 10^6^ kg in water depth 20 m. Under T2-II-1 earthquake action, the curvature changed significantly as the sea ice mass increased. The curvature reaches the maximum when the sea ice mass ranges from 1 × 10^7^ kg to 5 × 10^7^ kg. Consequently, the sea ice mass exerts a significant effect upon the maximum curvature of the structure, and maximum curvature mainly appears when ice mass varies range from 5 × 10^6^ kg to 5 × 10^7^ kg under near- and far-field seismic actions. Effects of sea ice on maximum curvature of the structure change as water depth varies.

**Figure 5:**
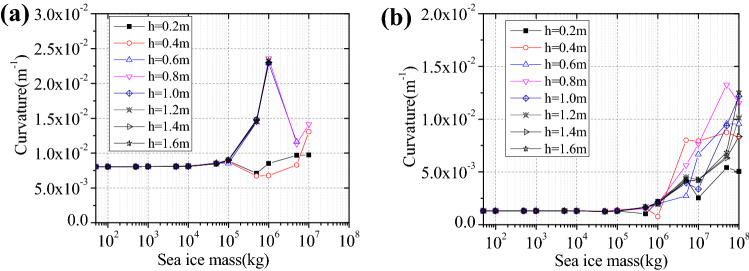
Maximum curvature of the tower bottom in different ice thicknesses: (**a**) T1-II-1; (**b**) T2-II-1.

The thickness of sea ice around the structure changes with both season and temperature, so its influences upon curvature at the structural bottom are investigated. The ice is set to be 0.2, 0.4, 0.6, 0.8, 1.0, 1.2, 1.4, and 1.6 m thick, and the water to be 15 m deep, as shown in Fig. 5.

As shown in Fig. 5, the influence of the ice thicknesses on maximum curvature is not distinct in the case of ice with mass lower than 1 × 10^6^ kg, and curvature begins to change significantly with the increasing ice mass under near- and far-field earthquake actions. Under T1-II-1 earthquake action, the maximum curvature is much larger than the ultimate curvature in the ice mass range from 1 × 10^6^ kg to 1 × 10^7^ kg when the ice thickness is greater than 0.4 m, which shows that the plastic failure has occurred to the structure. Under T2-II-1 earthquake action, the curvature increases with the ice thickness increasing when the ice mass is greater than 1 × 10^6^ kg, and the maximum curvatures are less than the ultimate curvature, which shows that the structure only produces plastic, but not damage.

**Figure 6:**
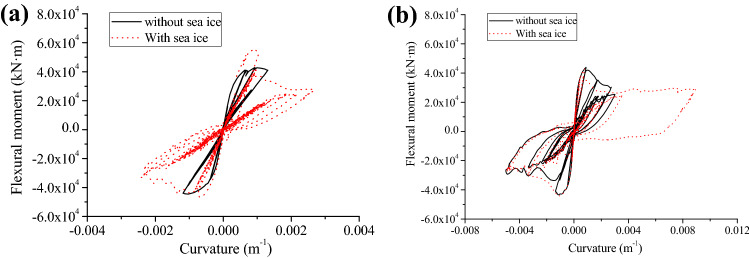
Bending moment and curvature hysteresis curves of the wind turbine tower bottom: (**a**) T1-II-1; (**b**) T2-II-1.

From the above analysis, we found that the most unfavourable ice mass is 1 × 10^7^ kg, and we set the ice mass and water depth to 1 × 10^7^ kg and 20 m, respectively. Then, the bending moment and curvature hysteresis curves of the tower bottom were investigated (Fig. 6).

**Figure 7:**
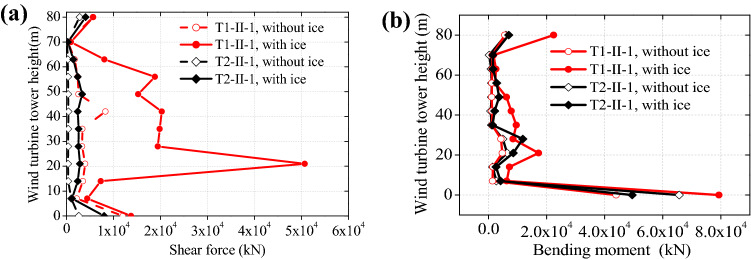
Influences of sea ice upon force in the structure: (**a**) shear force; (**b**) bending moment.

As shown in Fig. 6, the bending moment and curvature hysteresis curves present different shapes with and without the ice influence, and the sea ice has a considerable effect upon hysteresis curves between curvature and bending moment. The hysteresis curves are in a more obvious inverted *S* shape than that when there is no ice, suggesting the wind turbine tower having decreased deformation and energy dissipation capacity. So, in seismic design, the thickness of walls or stiffening ribs needs to be enlarged for improving structural ductility and seismic performance. Next, by investigating influences of the sea ice upon force in the structure, the position liable to plastic failure can be determined, as shown in Fig. 7.

Figure 7 shows that, there is greater force (bending moment and shear force) in the structure under ice influences than there are no ice influences under far- and far-field seismic actions, and the maximum internal force is found at the structural bottom. Affected by the ice, the action point of the sea ice also bears greater bending moment and shear force as well, which separately increased by 374.4% and 221.7%. The reason is that the ice, as a part of the tower system, moves with the tower in response to seismic actions, which increases horizontal seismic force on the structure, so design of wind turbine towers needs to be attached more importance to. Therefore, in seismic design of a wind turbine tower, carrying out anti-seismic check calculations on structural bottom and the action point of sea ice is a necessity; it is also necessary to add a stiffener in this position to ensure the structural safety.

**Figure 8:**
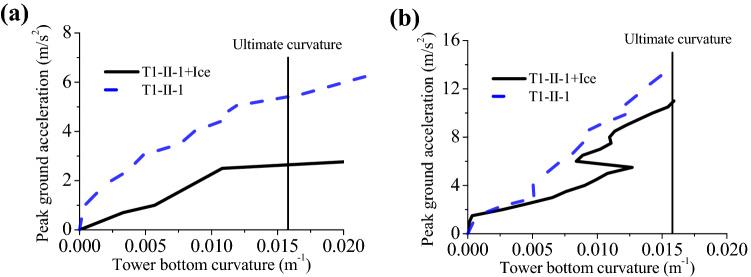
Change laws of the curvature with the PGA: (**a**) T1-II-1; (**b**) T2-II-1.

Additionally, for ascertaining influences of sea ice on the earthquake vulnerability of the wind turbine, peak ground acceleration (PGA) was adopted as an index for seismic intensity, and the index for structural damage was curvature at tower bottom. Then we could obtain the vulnerability curve of the structure. First, change laws of curvature with PGA are shown in Fig. 8. The average thickness of the ice is 0.6 m, and the water depths is 15 m.

As Fig. 8 shows, curvature responses of the structure are significantly different with and without ice influence under the same PGA. Especially under the far-field earthquake action, the curvature increases significantly affected by the ice. The curvature is smaller under the far-field seismic actions than under near-field actions. For example, under T1-II-1 earthquake action, the structure is damaged under ice influences when the PGA is about 2.5 m/s^2^; the structure is damaged in the presence of ice influences when the PGA is about 10 m/s^2^ under T2-II-1 earthquake action. Therefore, the wind turbine tower may be damaged due to the different types of seismic waves even under the same magnitude earthquake in the seismic design.

## Shaking-table tests

Considering that shaking-table tests could simulate dynamic behaviours of wind turbines under seismic actions^25,26^, we conducted the shanking table tests for verifying rationality of the simplified model.

### Test equipment and test table

**Figure 9:**
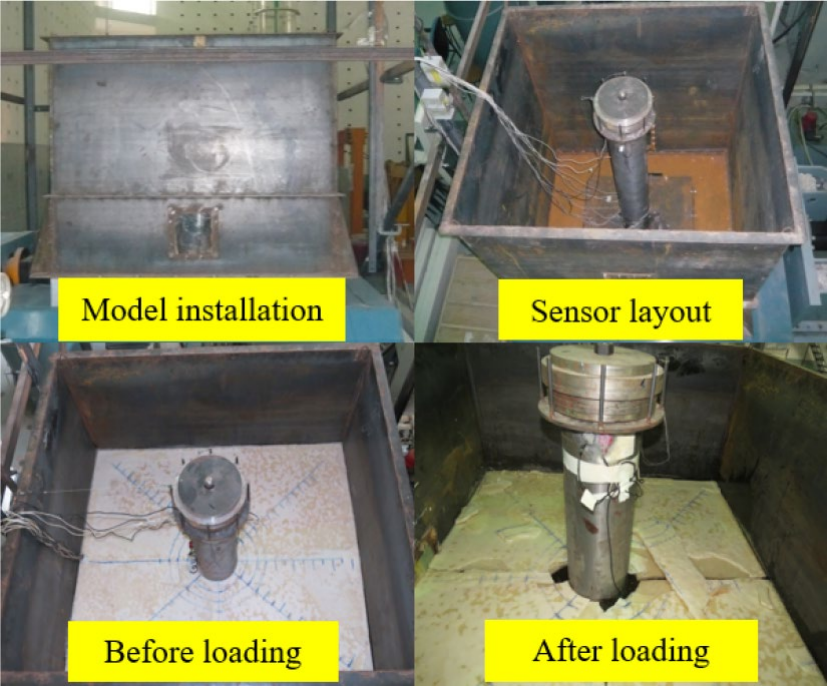
Test model.

The parameter information of the shaking-table equipment can be seen in reference^22^. To simulate the ice and water, a water tank was made. The test model is fixed inside the tank. The length of the tank is 2.0 m, the width is 1.3 m, and the height is 1.5 m. Considering that the shaking table measures 1.5 m × 1.5 m, the similarity coefficient is $\lambda_{l}$ = 1:50. Therefore, the height of the test model is 1.6 m, as shown in Fig. 9.

Before derivation of similar formulas, we make the following assumptions:

1. Water, ice, and structure following a same similarity relation.
2. Ensuring undistorted gravity of the structure, and adding mass blocks to the model top as mass of the upper structure on the basis of no influence on structural stiffness.
3. Controlling parameters of applied dynamic load for satisfying performance requirements of the loading device.

In the shaking-table tests on interplays among ice, structure, and water, it is ideal when the model and prototype structures satisfy similarity relationships in terms of gravity, inertial force, and resilience; whereas, there are strict requirements on density and elasticity modulus of structural materials. Hence, researchers generally use a similarity law of elastic force-gravity when performing the tests, to ensure consistency of the Cauchy constant and Froude constant in prototype and tested structures^27^.

The similarity law for elastic force-gravity was followed according to the abovementioned design principles for similarity ratios using non-prototype materials. In terms of the scaling law, we obeyed Buckingham-π theorem for prototype and model structures^28^. Because this model is designed to be at the elastic response stage, materials are supposed to be consistent with fundamental assumptions in the general elasticity theory as much as possible. So, we can use steel for similar model design. Equation (7) is a dynamic equilibrium equation at x direction in the elastic range.

7 $$ \frac{{\partial \sigma_{x} }}{\partial x} + \frac{{\partial \tau_{xy} }}{\partial y} + \frac{{\partial \tau_{xz} }}{\partial z} + \gamma x = \rho \frac{{\partial^{2} u}}{{\partial t^{2} }} $$

where $\sigma_{x}$, $\tau_{xy}$, $u$, and $\gamma$ separately represent stress at x direction, shear stress, displacement, and unit weight.

Through integral operation, Eq. (8) is attained.

8 $$ 0.5t^{2} \left( {\frac{{\partial \sigma_{x} }}{\partial x} + \frac{{\partial \tau_{xy} }}{\partial y} + \frac{{\partial \tau_{xz} }}{\partial z} + \gamma x} \right) + Ct + D = \rho u\;\left( {C = D = 0} \right) $$

Equations (9) and (10) are attained by substituting similarity constants.

9 $$ 0.5t^{{\prime}{2}} \lambda_{t}^{2} \left[ {\frac{{\lambda_{\sigma } }}{\lambda L}\left( {\frac{{\partial \sigma_{{x^{\prime}}} }}{{\partial x^{\prime}}} + \frac{{\partial \tau_{{xy^{\prime}}} }}{{\partial y^{\prime}}} + \frac{{\partial \tau_{{xz^{\prime}}} }}{{\partial z^{\prime}}}} \right) + \lambda_{\gamma } \gamma_{{x^{\prime}}} } \right] = \lambda_{\rho } \lambda_{u} \rho^{\prime}u^{\prime} $$

10 $$ 0.5t^{{\prime}{2}} \left[ {\left( {\frac{{\partial \sigma_{{x^{\prime}}} }}{{\partial x^{\prime}}} + \frac{{\partial \tau_{{xy^{\prime}}} }}{{\partial y^{\prime}}} + \frac{{\partial \tau_{{xz^{\prime}}} }}{{\partial z^{\prime}}}} \right) + \frac{{\lambda_{\gamma } \lambda_{l} }}{{\lambda_{\sigma } }}\gamma_{{x^{\prime}}} } \right] = \frac{{\lambda_{\rho } \lambda_{u} \lambda_{L} }}{{\partial_{\tau }^{2} \lambda_{\sigma } }}\rho^{\prime}u^{\prime} $$

where $\lambda_{t}$, $\lambda_{\gamma }$, $\lambda_{\sigma }$, and $\lambda_{\sigma }$ are similarity constants of time, unit weight, stress, and length, respectively.

By comparing Eq. (10) with Eq. (11) below,

11 $$ 0.5t^{{\prime}{2}} \left[ {\left( {\frac{{\partial \sigma_{{x^{\prime}}} }}{{\partial x^{\prime}}} + \frac{{\partial \tau_{{xy^{\prime}}} }}{{\partial y^{\prime}}} + \frac{{\partial \tau_{{xz^{\prime}}} }}{{\partial z^{\prime}}}} \right) + \gamma_{{x^{\prime}}} } \right] = \rho^{\prime}u^{\prime} $$

these relations can be attained: $\lambda_{\sigma } = \lambda_{\gamma } \lambda_{l}$ and $\lambda_{\sigma } = \frac{{\lambda_{\rho } \lambda_{u} \lambda_{l} }}{{\lambda_{\tau }^{2} }}$. Based on the elasticity law, there are $\lambda_{\sigma } { = }\lambda_{E} \lambda_{\varepsilon }$ and $\lambda_{u} = \lambda_{l} \lambda_{\varepsilon }$. These relations can then be obtained: $\lambda_{\varepsilon } = \frac{{\lambda_{\gamma } \lambda_{l} }}{{\lambda_{E} }} = \frac{{\lambda_{\rho } \lambda_{g} \lambda_{l} }}{{\lambda_{E} }}$ and $\lambda_{t} = \lambda_{l} \sqrt {\frac{{\lambda_{\rho } }}{{\lambda_{E} }}}$. Due to dimensionless nature of Poisson's ratio and strain, $\lambda_{\varepsilon }$ = 1 and $\lambda_{\upsilon }$ = 1.

Because of limited equipment size in the tests, boundary conditions of water are restricted. Under the condition, we set a similarity constant $\lambda_{l}$ = 0.05 to more favourably simulate infinite boundary conditions of water. We performed dimensional analysis to derive similarity relationships (Table 2).

**Table 2:** Similarity coefficients for the test model.

Physical quantity	Dimension	Similarity coefficient
Length	L	$\lambda_{l}$ = 0.02
Displacement	L	$\lambda_{u}$ = $\lambda_{l}$ = 0.02
Area	L^2^	$\lambda_{A} = \lambda_{l}^{2}$ = 0.0004
Elasticity modulus	FL^−2^	$\lambda_{E}$ = 6.2
Stress	FL^−2^	$\lambda_{\sigma }$ = $\lambda_{E}$ = 6.2
Strain	/	$\lambda_{\varepsilon }$ = $\lambda_{\sigma }$/$\lambda_{E}$ = 1
Density	FL^−4^T^2^	$\lambda_{\rho }$ = 3.12
Time	T	$\lambda_{t} = \lambda_{l} \sqrt {\lambda_{\rho } /\lambda_{E} }$ = 0.030
Acceleration	LT^−2^	$\lambda_{a}$ = 0.41

### Ice material selection

Paraffin is used to simulate the sea ice, and the compression test is conducted to determine the physical and mechanics parameters of the paraffin. After being cut into little cylindrical blocks, the paraffin is crushed under compression. We determined the stress and strain of each sample (Fig. S4), and calculated the elastic modulus of each sample, as shown in Table 3.

**Table 3:** Elastic moduli of various samples.

No.	1	2	3	4
Elastic modulus (MPa)	100.54	84.66	156.94	117.65

As shown in Table 3, the mechanical behaviour and elastic modulus of the fourth sample are similar to the sea ice. Thus, the fourth sample is used to simulate the sea ice.

### Experimental scheme

**Figure 10:**
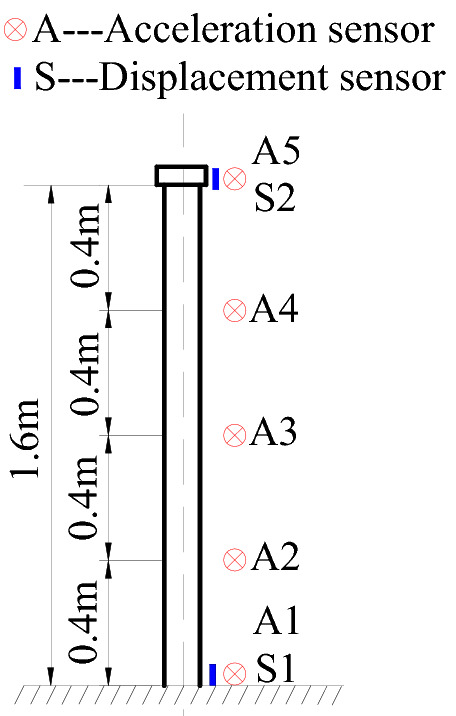
Sensor placement.

In the shaking-table test, five acceleration sensors and two displacement sensors are used to measure the displacement and acceleration for the test model. The sensor model can be seen in reference^22^, and the sensor placement is shown in Fig. 10.

### Analysis of test results

**Figure 11:**
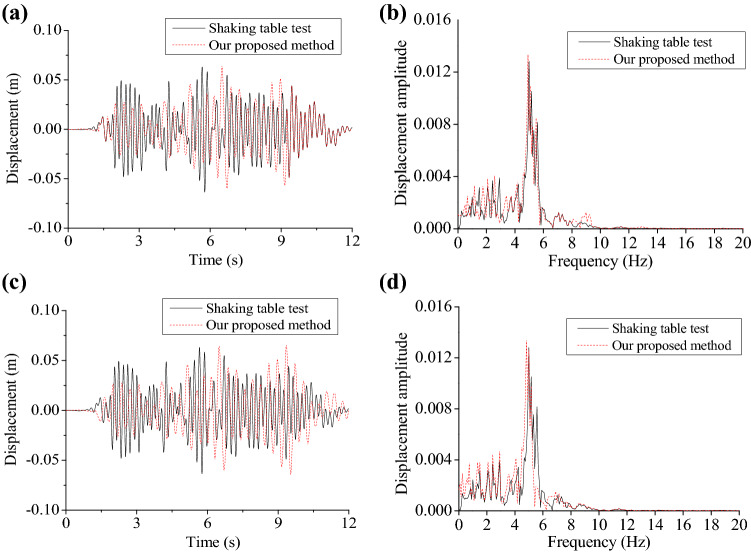
Time histories and Fourier spectra for displacement in the presence and absence of ice influences: (**a**) displacement influenced by ice; (**b**) Fourier spectrum influenced by ice; (**c**) displacement without ice influence; (**d**) Fourier spectrum without ice influence.

**Figure 12:**
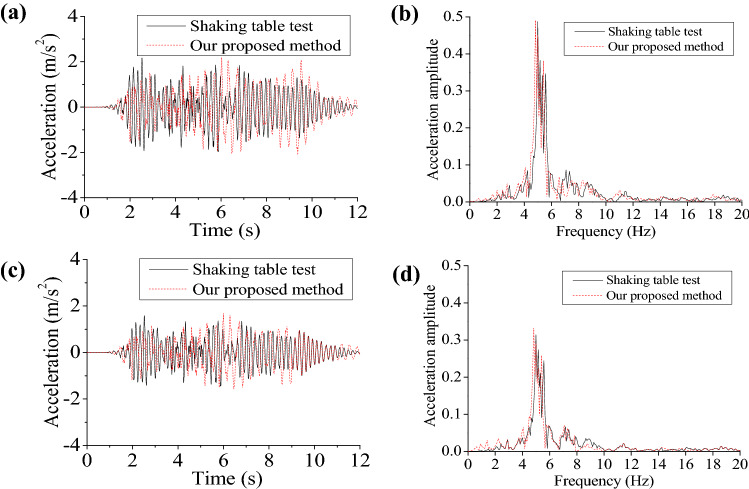
Time histories and Fourier spectra for acceleration in the presence and absence of ice influences: (**a**) acceleration influenced by ice; (**b**) Fourier spectrum influenced by ice; (**c**) acceleration without ice influence; (**d**) Fourier spectrum without ice influence.

To testify that the calculation results are accurate, T1-II-1 seismic waves were taken as examples, with peak acceleration is set as 1 m/s^2^. We then compared time histories and Fourier spectra for displacement and acceleration at the model top, as shown in Figs. 11 and 12.

As shown in Figs. 11 and 12, by comparing displacement and acceleration in terms of their time histories, the time histories calculated in response to seismic actions are in good agreement with test data; time histories for displacement and acceleration are found to have consistency in change laws with the test results, with the largest deviation of 16% around in response to seismic actions in the presence and absence of ice influences. Comparison of Fourier spectra for acceleration and displacement discovered that test results are agreed well with calculated results, particularly in the part of high frequencies. Whereas, relative to test results, there are larger calculated displacement and acceleration. This is because the model is elasto-plastic, while the approach proposed is to some extent simplified. In general, time histories and Fourier spectra for calculated displacement and acceleration using the proposed approach conform well to results of shaking-table tests, proving both accuracy and reliability of the approach proposed. Besides, effects of sea ice upon displacement and acceleration of the structure under sine waves (1 and 3 Hz) are investigated (Figs. S5 and S6). Time histories and Fourier spectra of displacement and acceleration at the top of the model under T2-II-1 seismic waves were also compared in Figs. S7 and S8.

**Figure 13:**
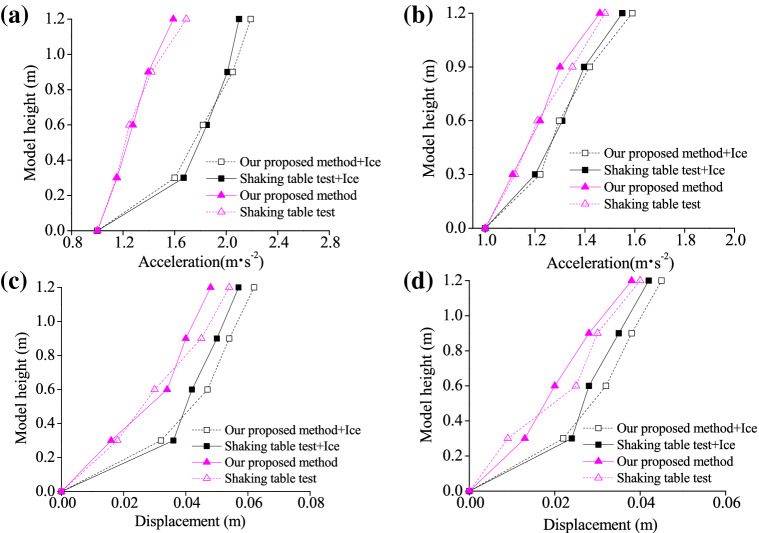
Peak accelerations and displacements along height direction of the model in the presence and absence of influences of sea ice: (**a**) T1-II-1 (acceleration); (**b**) T2-II-1(acceleration); (**c**) T1-II-1 (displacement); (**d**) T2-II-1 (displacement).

Finally, calculated peak acceleration and displacement along height direction of the model were compared in the presence and absence of influences of sea ice (Fig. 13).

As shown in Fig. 13, the peak displacement and acceleration along height direction of the model under seismic actions have test results conforming to results calculated using the proposed approach. In addition, only certain deviations are discovered on specific conditions. The peak acceleration has the largest difference of 12.18% under El Centro actions; while that for the peak displacement is 14.20% under T1-II-1 actions. Peak displacement and acceleration solved by the method proposed along the model height show distribution laws agreed with test data on the whole, further verifying both accuracy and reliability of the proposed method. Besides, it is found that displacement and acceleration under influences of sea ice are significantly larger than those in the absence of such influences. This indicates that sea ice has influences that cannot be ignored upon displacement and acceleration in seismic design of offshore structures.

## Conclusion

The simplified model for dynamic interactions among ice, structure, and water was proposed for studying effects of sea ice upon seismic behaviours of the wind turbine tower. To verify rationality of our proposed simplified model, we conducted shaking-table tests. The following conclusions were drawn:

1. The added mass and springs of certain damping and stiffness were used to simulate the effect of the ice on wind power tower, and a simplified calculation model for the wind turbine tower, ice, and water dynamic interaction under seismic load was established. This obviated the need to solve complicated non-linear equations and reduced the computational burden to a significant extent.
2. When the ice mass is within a certain range, it exerted a significant influence upon curvature at the bottom of the tower, which could reflect the plastic deformation of the structure. Under effects of sea ice, the structure exhibited decreased deformation and energy dissipation capacity; hence, it needs to enlarge thickness of tower walls or stiffening ribs for improving ductility and seismic performance of the structure. The wind turbine tower may be damaged due to different seismic waves even under same magnitude earthquakes in the seismic design.
3. The tower bottom and action point of sea ice showed significantly increased maximum bending moment and shear force; thus, it was deemed necessary to conduct anti-seismic design check-calculations on the tower bottom and action point under influences of unfavourable sea ice mass in seismic design of the structure. Based on the design principle of the similarity ratio, paraffin was used to simulate sea ice and shaking-table tests were conducted for verifying accuracy of the simplified model proposed, and calculated data were within accepted tolerances.

## Supplementary Information

Supplementary Information 1.
