# Supplementary material for: Seismic behaviour analysis of a wind turbine tower affected by sea ice based on a simplified model
Source: Sci Rep. 2021 Mar 24;11:6714. doi: 10.1038/s41598-021-86142-0 (PMC7991629; doi:10.1038/s41598-021-86142-0)
Supplement: Supplementary file 1 — Supplementary Information 1. [file 41598_2021_86142_MOESM1_ESM.docx]

# Supplementary information

Seismic behaviour analysis of a wind turbine tower affected by sea ice based on a simplified model

Shuai Huang^1^*, Qingjie Qi^2^, Shufeng Zhai^3^, Wengang Liu^2^, Jianzhong Liu^2^

1.National Institute of Natural Hazards, Ministry of Emergency Management of China, Beijing 100085, China

2.China Coal Research Institute, Beijing 100013, China

3.Institute of Disaster Prevention, Hebei 065201, China

[huangshuai3395@163.com](mailto:correo_electrónico@autor.correspondiente)

## Supplementary Figure

(a)

(b)

Supplementary Figure S1: The simplified calculation model: a) Geometric figure; b) Schematic diagram of simplified model.

Supplementary Figure S2: Croteau ice force model.

Supplementary Figure S3: Constitutive model of the wind turbine tower.

Supplementary Figure S4: Stress-strain data.

(b)

(a)

(c)

(d)

Supplementary Figure S5: (a) Acceleration at the model bottom (1Hz); (b) Acceleration at the model top(1Hz); (c) Acceleration at the model bottom (3Hz); (d) Acceleration at the model top (3Hz).

(b)

(a)

(a) 1 Hz (b) 3 Hz

Supplementary Figure S6: (a) Displacement under sinusoidal input at 1 Hz; (b) Displacement under sinusoidal input at 3 Hz. The effect of the sea ice on the acceleration and displacement is not so obvious at low frequency (1 Hz) while the acceleration and displacement vary obvious at high frequency (3 Hz) ( Figure S5, Figure S6), which shows that the high frequency composition should be mainly considered in the dynamic interaction of the structure and the ice. Also, we could see that the effect of the sea ice on the displacement is greater than acceleration at the high frequency. Affected by the sea ice, the displacement increases 415% while the acceleration only increases 42% at the high frequency. The effect of the sea ice on the acceleration is greater than displacement at the low frequency. In addition, the acceleration and displacement of the test model top are greater than those of the test model bottom, which shows that the dynamic amplification effect is generated along the height of the structure.

(b)

(a)

(d)

(c)

Supplementary Figure S7: Time history of displacement and Fourier spectrum with and without ice influence under T2-II-1 action: (a) Displacement influenced by ice; (b) Fourier spectrum influenced by ice; (c) Displacement without ice influence; (d) Fourier spectrum without ice influence.

(a)

(b)

(c)

(d)

Supplementary Figure S8: Time history of acceleration and Fourier spectrum with and without ice influence under T2-II-1 action: (a) Acceleration influenced by ice; (b) Fourier spectrum influenced by ice; (c) Acceleration without ice influence; (d) Fourier spectrum without ice influence.
